# Is there a clinical benefit of additional tension band wiring in plate fixation of the symphysis?

**DOI:** 10.1186/s12891-017-1418-3

**Published:** 2017-01-25

**Authors:** Myung-sik Park, Sun-Jung Yoon, Seung-min Choi, Kwanghun Lee

**Affiliations:** Department of Orthopedic Surgery, Chonbuk National University Hospital, Research Institute of Clinical Medicine of Chonbuk National University, Biomedical Research Institute of Chonbuk National University Hospital, Jeonju, South Korea

**Keywords:** Tension band wiring, Plate fixation, Traumatic symphysis pubis diastasis, Pelvic ring injury

## Abstract

**Background:**

The purpose of this study was to determine whether additional tension band wiring in the plate for traumatic disruption of symphysis pubis has clinical benefits. Therefore, outcomes and complications were compared between a plate fixation group and a plate with tension band wiring group.

**Methods:**

We retrospectively evaluated 64 consecutive patients who underwent open reduction and internal fixation of the symphysis pubis by using a plate alone (*n =* 39) or a plate with tension band wiring (*n =* 25). All the patients were followed up for a minimum of 24 months (mean, 34.4 months; range, 26–39 months). Demographic characteristics, outcomes, movement of the metal works, complications, revision surgery, and Majeed functional score were compared.

**Results:**

Significant screw pullout was relatively significantly more frequently found in the plate fixation group than in the plate with tension band wiring group (*P =* 0.009). In terms of the overall rate of all-cause revision surgery, including significant loosening, symptomatic hardware, and patient-requested hardware removal during follow-up period, the plate with tension band wiring group showed a significantly lower rate.

**Conclusion:**

Tension band wiring in combination with a symphyseal plate showed better radiological outcomes, a lower incidence of hardware loosening, and a lower rate of revision surgery than plate fixation alone. This technique would have some potential advantages in terms of avoiding significant movement of plate, symptomatic hardware failure, and revision surgery.

## Background

Open reduction and internal fixation (ORIF) using a plate and screws facilitates accurate reduction and is now the most reliable method of stabilization for disrupted pubic symphysis [1, 2]. Although plate fixation has a lower complication rate than wiring or screw fixation alone, and has become the popular method of symphyseal fixation, it has shown different results depending on the type of plate used [3, 4]. Several authors reported that the rates of hardware failure, loss of reduction, and revision rates range from 12 to 31%, from 7 to 24%, and from 3 to 9%, respectively [4–8]. Results are largely inconsistent, thus the varying reports about plate fixation of the pubic symphysis. Surgical complications after plate fixation are frequent and include fixation failure, infection, rewidening of symphyseal width, movement of plate-screw construct, and soft tissue irritation, with the latter two being the most common causes of revision surgery. This revision could cause distress for patients and surgeons.

A mechanical testing of anterior stabilization in pubic symphysis separation has been reported that tension band wiring could resist vertical loading [9]. The combination of plate and tension band wiring would reduce implant failures, including movement that cause plate-screw construct breakage, soft tissue irritation, and revision surgery, better than plate fixation alone. In our previous study, we reported that tension band wiring in plate fixation is an applicable technique for traumatic rupture of the symphysis pubis [10].

This study examined a combination of pubic symphysis plate and tension band wiring in ORIF of traumatic pubic symphysis diastasis. In addition, we investigated the outcomes of the use of a plate with tension band wiring in comparison with those of plate fixation alone for disrupted pubic symphysis.

## Methods

Between March 2009 and March 2013, 64 patients with pubic symphysis rupture underwent ORIF with a plate alone or a plate with tension band wiring. All the patients were followed up for a minimum of 24 months (mean, 34.4 months; range, 26–39 months). Of the patients, 54 were male and 10 were female, with a mean age of 42.7 years (range, 16–74 years). We had institutional review board approval for this retrospective study.

At the time of injury, all the patients were evaluated and treated in accordance with advanced trauma life support protocols. This was followed by standardized imaging of the pelvis, including anteroposterior (AP), inlet, and outlet plain radiography and computed tomography. Injury radiographs were classified by using the Tile [11] and the orthopedic trauma association (OTA) classification systems [12].

**Fig. 1:**
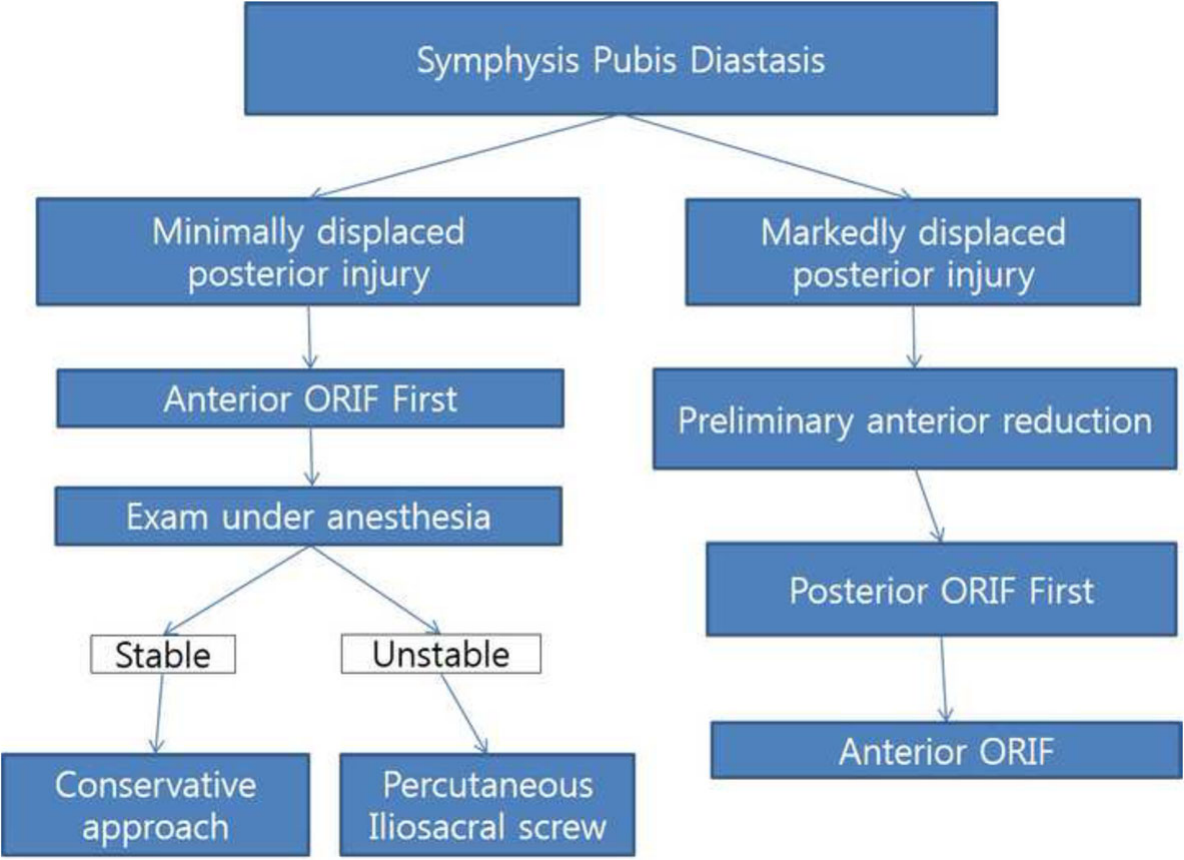
Flowchart showing the treatment process. A flowchart showing the decision-making process for anterior and/or posterior fixation of a pelvic ring injury with symphysis pubis diastasis

Our indication for anterior plate fixation included open-book injury with a diastasis of the pubic symphysis of >25 mm. Posterior fixation was additionally performed if the displacement extended all the way through the posterior part of the SI joint or sacral fracture, and complete posterior arch disruption. Otherwise, stabilization was performed in accordance with the operating surgeon’s preference and decision-making process (Fig. 1). Patients with open injuries or associated acetabular fractures, and patients definitively managed with additional external pelvic fixator devices were excluded.

All the cases of ruptured symphysis pubis were approached through a midline vertical rectus splitting with the Pfannenstiel skin incision. In vertically unstable fractures, a preliminary anterior reduction was achieved first, and then the posterior ring was reduced and fixed, followed by application of the definitive anterior plate. Anterior fixation was achieved by using a plate and screws (C&S Medical, Seoul, South Korea) with the aim of reducing all ruptured pubic symphysis anatomically. Typically, a single six- or four-hole plate and 4.5-mm screws were used, but actual fixation was dependent on the associated injury pattern. If the injury involved the pubic rami, then the plate length was extended and the number of screws was increased.

**Fig. 2:**
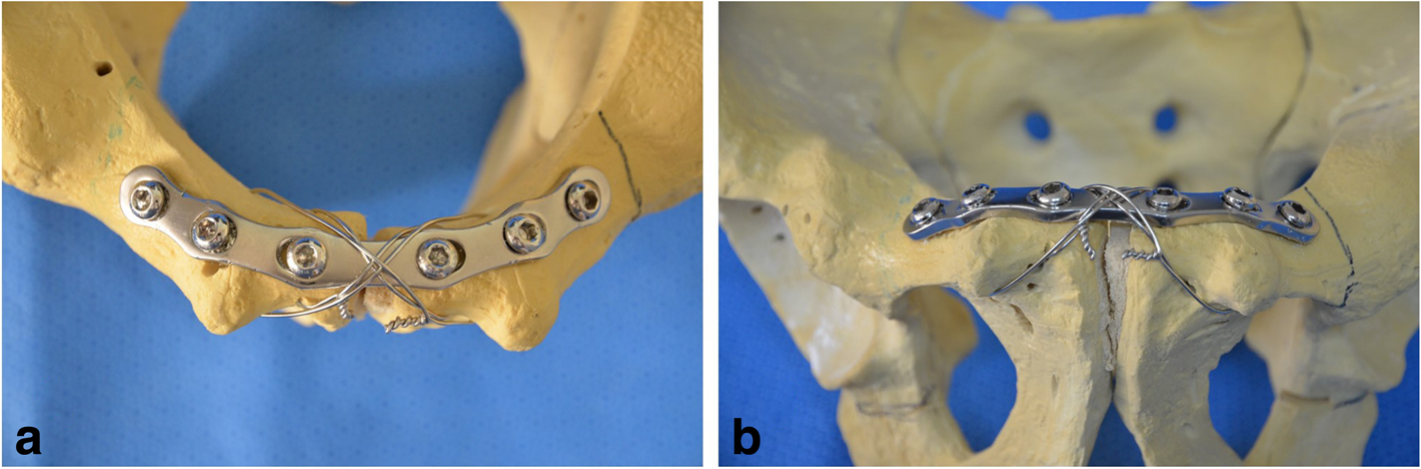
Tension band wiring technique over the plate. Tension band wiring technique over the plate fixation of disrupted symphysis pubis. Inlet view (**a**) and outlet view (**b**)

From January 2012, a policy change was introduced regarding augmentation of one or two figure-of-8 wires over the plate in a tension band fashion. To study the consequences of this change, we divided the patients into 2 groups, the plate fixation group and the plate fixation with tension band wiring group. During the study, 39 patients underwent plate fixation only, and 25 patients underwent symphysis pubis plating with tension band wiring. After the ORIF was finished, a Cobb’s elevator or a malleable retractor can be used to protect the structures in the Retzius space during the making of holes for wire passage in the body of the pubis with a drill. As described previously [10], as an alternative method, the wire could be passed through the medial corner of the obturator foramen. However, this method requires more dissection of muscle attached to the pubic body and rami. Drill holes on the pubic body were better than passing through the medial corner of the obturator foramen to decrease the risk of damaging neurovascular bundles. In addition, contrary to opinion among some surgeons that making a hole in the pubic body in elderly patients with osteoporotic bone quality could have risks of fractures to the rami during tightening of the wire, we encountered no such complications when using the technique. Generally, two figure-of-8 tension band cerclage wires (1.25 mm in diameter; Synthes) were augmented over the plate through drilling holes for wires on the pubic body after the plate fixation (Fig. 2). The additional tension-band wiring procedure generally took 10 min. For associated posterior injuries, alternative approaches were used, including the anterior surgical approach and reduction of sacroiliac joint dislocation, or a posterior approach and fixation for displaced or complete posterior injuries. Supplementary posterior ring fixation was performed in 20 patients to stabilize posterior injuries with displaced or comminuted sacral fractures or sacroiliac joint fracture subluxations. Percutaneous iliosacral screw fixation was usually the preferred technique. Open reduction and anterior plating were performed for only select cases when closed reduction was not possible or when an anterior approach to the innominate bone was required for another injury.

After surgery, toe-touch weight bearing on the side of the hemipelvic injury were allowed for 6 weeks. Partial weight bearing to 50% was increased for 12 weeks, and full weight bearing was started after 12 weeks.

A retrospective review of medical charts and radiographs was conducted to analyze and compare clinical and radiographic outcomes. Preoperative data from the 2 groups, including patient demographic characteristics, injury mechanism, fracture classification, and associated injuries, were compared. Radiographic follow-up was performed before primary treatment, after surgery, and during the follow-up period. Radiographic changes and information on revision surgery were classified into an immediate postoperative period (<4 weeks), early/midterm (3–12 months), and late follow-up period (13–24 months) to specify complications. Immediate postoperative and follow-up AP radiographs at 12 months were reviewed for screw loosening, metal breakage, and recurrent widening of the symphysis pubis. As described previously [10], revision surgery was defined as any surgical procedure that involved an open treatment to address a hardware failure, including infection or symptomatic hardware (defined as discomfort or irritation). Implant removal was performed only in symptomatic patients or also in asymptomatic patients with gross radiographic widening/loosening. The degree of the loss of reduction of the pubic symphysis is measured based on the gap of the upper margin of the symphyseal width (the distance between the two sides of the symphyseal joint of the pubis) on AP pelvic radiographs. The last follow-up AP radiography was performed to evaluate for subjective radiographic loss of fixation; screws were considered loosened if backing out, separation between the screw head and the plate, or a distinct radiographic halo (lysis) around the screw threads was observed. Any of these changes on follow-up radiographs from the immediate postoperative films were noted. Significant screw pullout or loosening indicated that half of the screw length escaped from its original position compared with that on the immediate postoperative radiograph. Recurrent diastasis was defined as a symphyseal widening of >10 mm on immediate postoperative radiographs during the follow-up period. Data of complications were collected in the early postoperative (<1 month) and follow-up phases during the study period.

Clinical and radiographic data were collected at the 3-month, 6-month, and 1-year follow-ups. Thereafter, the patients were examined at 1-year intervals. The functional outcome at 2 years after operation was measured by using a scoring system described by Majeed [13].

The Mann–Whitney *U* test was used to identify the differences between the two groups. Categorical variables were analyzed by using the Fisher exact test. A *P* value of <0.05 was considered significant.

## Results

### Postoperative follow-up

Preoperative data from the two groups were compared, and the study showed no significant differences in all preoperative variables between the two groups (Table 1). All the 44 patients who underwent anterior stabilization alone without posterior fixation had injuries characterized as partially stable sacroiliac joint disruption or minimally displaced sacral fractures. Twenty patients had additional posterior pelvic fixation, 18 of whom were treated with one or two 6.5- or 8.0-mm iliosacral screw fixation. For OTA type C unstable posterior pelvic injury, three patients had an ORIF for the sacroiliac joint fracture dislocation and two had double anterior plating for the sacroiliac joint, one of whom had an additional iliosacral screw fixation.

**Table 1:** Comparison of preoperative data between the groups

Variable	Plate only (*n =* 39)	Plate with tension band wiring (*n =* 25)	*P*
Mean age (range), yr	41.9 (16–74)	47.9 (20–74)	0.132
Sex, n (%)			0.292
Male	31 (79.4)	23 (92)	
Female	8 (20.5)	2 (8)	
Associated injury, n (%)			0.746
Major	11 (28.2)	8 (32)	
Minor	28 (71.8)	17 (68)	
Fracture pattern, n (%)			0.171
AO/OTA 61-B	31 (79.4)	16 (64)	0.246
AO/OTA 61-C	8 (20.5)	9 (36)	

Major injuries including head, chest, abdominal, spinal or vascular injury, need intervention

Minor injuries include lower or upper limb injuries

Surgery-related variables from the two groups were compared in terms of the number of posterior fixations and reduction in the quality of pubic symphysis, which showed no statistical significant difference, but the number of screws for anterior fixation showed a significant difference (Table 2). The number of screws was smaller in the plate with tension band wiring group than in the plate-only group (median, 5 vs. 7; *P* < 0.001). The difference in posterior fixation between the two groups was not significant (*P =* 0.917).

**Table 2:** Surgery-related variables

	n (%)		
	Plate only (*n =* 39)	Plate with tension band wiring (*n =* 25)	*P*
Posterior stabilization, n (%)	12 (30)	8 (32)	0.917
Reduction quality, n (%)			
Satisfactory	35 (90)	23 (92)	0.93
Unsatisfactory	4 (10)	2 (8)	
Numbers of screws for anterior plating (median)	7	5	<0.001

In the plate fixation group, postoperative complications included fixation failure in 4 patients (10.3%), of whom 3 (7.7%) underwent revision surgery. One patient with fixation failure did not require further surgery because of a relatively good functional outcome despite the loss of fixation. In the plate with tension band wiring group, no early postoperative complication occurred.

### Early/Midterm follow-up

**Fig. 3:**
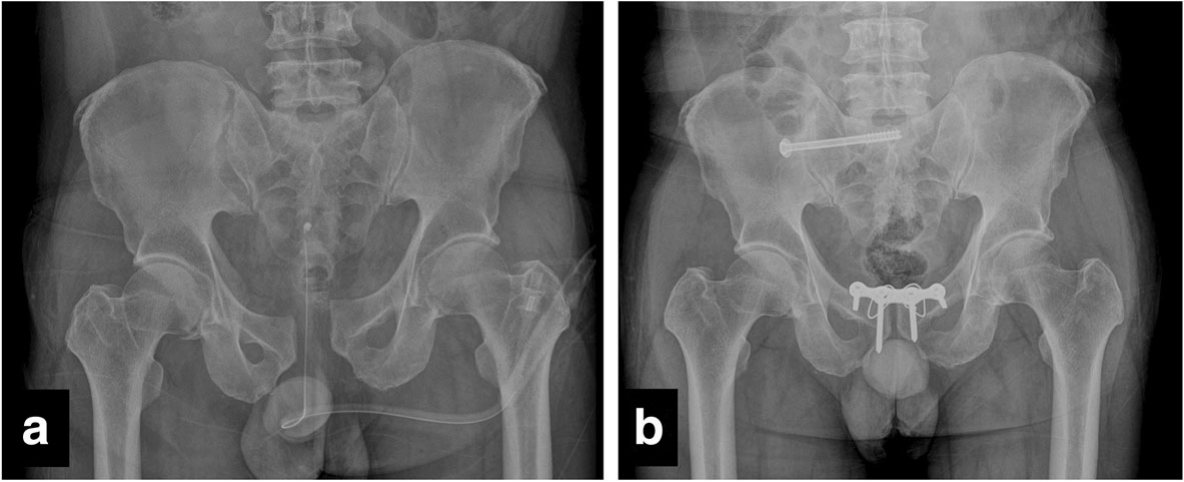
Plain pelvis radiograph. **a** Preoperative image of a 36-year-old male patient after pelvic ring injury (61-B1). **b** A follow-up image taken 1 year after surgery, showing anterior fixation with plate and screw fixation with tension band wiring and posterior fixation with an iliosacral screw for a right sacroiliac joint disruption

Widening of the symphyseal width (≥10 mm) was observed on the postoperative radiographs of 12 patients (30.7%), and the difference from that on the preoperative radiograph was significant (*P* = 0.036). Six of the patients were associated with screw pullout and underwent revision surgery. Significant screw pullout was relatively more frequently found in the plate-only group than in the plate with tension band wiring group, with a significant difference (*P* = 0.009). Seven of the patients underwent revision surgery. During the follow-up period, one patient (4%) had recurrent widening of the pubic symphysis at the first postoperative visit but did not require further surgical procedure because the functional outcome was good (Fig. 3).

### Late follow-up

All the patients were followed up for a minimum of 24 months (mean, 34.4 months; range, 26–39 months). The mean symphyseal width was smaller in the plate with tension band wiring group than in the plate-only group during the 1-year follow-up period. The symphyseal width was narrower and more stable in the 3-month postoperative assessment in the patients in the plate with tension band wiring group than in those in the plate-only group, whose width had gradually increased by this time. This result was found to be statistically significant at *P* < 0.05 (Table 3).

**Table 3:** Complications

	n (%)			
Complication	Plate only (*n =* 39)	Plate with tension band wiring (*n =* 25)	*P*	OR (95%CI)
Early postoperative complication (<4 weeks)				
Superficial wound infection	0	0		
Fixation failure/revision surgery	4 (10.3)	0		
Changes in plate-screw construct during the early/midterm follow-up period (4–12 months)				
Screw breakage	8 (20.5)	4 (16)	0.751	1.3 (0.36–5.08)
Significant screw pullout or loosening	15 (38.4)	2 (8)	0.009	1.7 (1.24–2.40)
Plate breakage	0	3 (12)	N/A	
Symphyseal width widening (≥10 mm) from its width at the immediate postoperative of 12 months	12 (30.7)	2 (8)	0.036	1.5 (1.13–2.21)
HWR or revision surgery due to any cause during late follow-up (13 months to 2 years)	6 (15.3)	1 (4)	0.231	1.4 (1.01–2.15)
All-cause overall revision surgery	10 (25.6)	1 (4)	0.039	1.66 (1.22–2.26)

Plate and tension band wiring was removed in a 26-year-old woman 15 months after the onset of an OTA type C fracture because she was planning to become pregnant and requested plate removal. The procedure was performed electively in combination with removal of two iliosacral screws.

In the all-cause overall rate of revision surgery, including hardware removal by any reason during follow-up, the plate with tension band wiring group showed a significantly lower frequency, including patient-requested surgery. The functional outcomes showed that functional recovery was better in the plate with tension band wiring group than in the plate-only group at 2 years after the operation (*P* = 0.029; Table 4). The mean Majeed pelvic score was 85.2 ± 8.5 in the plate with tension band wiring group and 75.5 ± 13.9 at 2-years’ follow-up.

**Table 4:** Clinical outcomes 2 years after fixation

		n (%)	
Radiologic Result	Plate only (*n =* 39)	Plate with tension band wiring (*n =* 25)	*P*
Excellent (>85)	15 (38.5)	16 (64)	0.029
Good (70–84)	10 (25.7)	8 (32)	
Fair (55–69)	10 (25.7)	1 (0.4)	
Poor (<55)	4 (10.3)	0 (0)	

## Discussion

The aim of this study was to compare the outcome of the plate with tension band wiring fixation technique with that of plate fixation alone in the treatment of traumatic pubic symphysis diastasis. In this study, additional tension band wiring over a symphyseal plate was quite effective for maintaining a plate and screw construct instrumented for diastasis of symphysis pubis, and our results demonstrated favorable radiographic outcomes. While the revision rate in the plate-only cases was similar to those reported in previous literatures, only one patient (4%) in the plate with tension band wiring group needed hardware removal.

Although anterior plating is the best preferred fixation technique for pubic symphysis disruption, the incidence and consequences of fixation failure according to the type of plate or fixation technique have been reported in the literatures to be up to 43% and thus remain a concern. The overall revision rate after open reduction and internal fixation with plates and screws were reported to range from 3 to 30% [14].

Our study is subject to several limitations. First, this was a retrospective study in which the complications were reviewed from the medical records, which may have led to minor complications being underreported. Second, this study had a small sample size and is potentially underpowered. A post hoc power analysis showed that with our sample in each group, our data had 48.4% power for detecting differences in overall revision surgery and a power of 68.7% for detecting differences in screw pullout. Third, bone quality and surgeon experience as potential confounders were not controlled in this study. Further investigation is needed to elucidate the advantages of additional tension band wiring over the plate by using biomechanical study. However, this technique is simple and adds little time or cost to the case, and seems to have some potential advantages to improve outcomes. Additional tension band wiring may be beneficial to maintain the plate and screw fixation in the treatment of diastasis of the symphysis pubis. Incidence of hardware removal was frequently low in the plate with tension band wiring group, including patient-requested surgery. The possible explanation of this result is that tension band wiring around the plate and screw construct may provide additional stability to prevent motion of the hardware after the union. It might affect the timing and clinical outcome of symptomatic hardware failure.

The plate with figure-of-8 tension band wiring would provide more sufficient stability than plate fixation alone. This technique might become an alternative option to minimize soft tissue injury and maximize stabilization of symphysis pubis diastasis. Well-designed comparative studies are needed to determine whether there is any benefit of tension band wiring over the symphyseal plate for patients.

## Conclusion

Tension band wiring in combination with a symphyseal plate showed better radiological outcomes, lower incidence of hardware loosening, and lower rate of revision surgery than plate fixation alone. This technique would have some potential advantages in terms of avoiding symptomatic hardware failure and revision surgery.
